# Prevalence of Oral Human Papillomavirus Infection among Youth, Sweden

**DOI:** 10.3201/eid1809.111731

**Published:** 2012-09

**Authors:** Juan Du, Cecilia Nordfors, Andreas Ährlund-Richter, Michal Sobkowiak, Mircea Romanitan, Anders Näsman, Sören Andersson, Torbjörn Ramqvist, Tina Dalianis

**Affiliations:** Karolinska Institutet, Stockholm, Sweden (J. Du, C. Nordfords, A. Ährlund-Richter, M. Sobkowiak, M. Romanitan, A. Näsman, T. Ramqvist, T. Dalianis);; Swedish Institute for Infectious Disease Control, Stockholm (S. Andersson);; and Örebro University Hospital, Örebro, Sweden (S. Andersson)

**Keywords:** HPV, human papillomavirus, oral HPV infection, cervical HPV infection, tonsillar cancer, base of tongue cancer, oropharyngeal cancer, cervical cancer, viruses, Sweden, young adults, youth, adolescents, *Suggested citation for this article*: Du J, Nordfors C, Ährlund-Richter A, Sobkowiak M, Romanitan M, Näsman A, et al. Prevalence of oral human papillomavirus infection among youth, Sweden. Emerg Infect Dis [serial on the internet]. 2012 Sep [*date cited*]. http://dx.doi.org/10.3201/eid1809.111731

## Abstract

Human papillomavirus (HPV) causes cervical, head, and neck cancers. We studied 483 patients at a youth clinic in Stockholm, Sweden, and found oral HPV prevalence was 9.3% and significantly higher for female youth with than without cervical HPV infection (p = 0.043). Most oral HPV types matched the co-occurring cervical types.

Human papillomavirus (HPV) causes cervical, head, and neck cancers (*1*). Recent reports show that oropharyngeal cancer, the head/neck cancer for which HPV infection is most common, is increasing (*2*). HPV vaccination with Gardasil (Merck, Whitehouse Station, NJ, USA) and Cervarix (GlaxoSmithKline, Brentford, UK) prevents cervical infection with HPV types 16 and 18, but less is known about oral HPV infection (*3*,*4*). To evaluate oral HPV prevalence before HPV vaccination of the public, we performed a study at a youth clinic in Stockholm, Sweden, where we previously reported high cervical HPV prevalence (70%) among female youth (*5*). We compared oral HPV prevalence in male and female youth visiting the clinic and studied oral HPV prevalence and type concordance in relation to cervical HPV infection.

## The Study

The study, performed during 2009–2011 with permission from the Stockholm Regional Ethical Committee, enrolled 408 female and 82 male youth, 15–23 years of age, who visited a large youth clinic in Stockholm (*5*). None had been vaccinated for HPV. In brief, ≈4,000 female and ≈800 male youth visit the clinic each year for birth control advice and treatment for sexually transmitted diseases (*5*). The low participation rate in our study was the result of periods of high workload with no enrollment, but when asked, most persons participated.

Oral samples from enrollees were obtained after 30 s of mouthwashing with 15 mL of 50% Listerine (Johnson & Johnson, New Brunswick, NJ, USA). Samples were stored at 4°C for a maximum of 3 days and then centrifuged at 6,000 × *g* for 10 min; the resulting pellet was stored at −20°C. DNA was extracted by using the Gentra Puregene Buccal Cell Kit (QIAGEN AB, Stockholm, Sweden) and dissolved in a 100-μL DNA hydration solution (provided with the kit).

Cervical samples (n = 180) were collected from female youth and prepared as described (*5*). A 10-μL aliquot for each sample was analyzed for 24 mucosal HPV types by using a Luminex-based multiplex assay, as described by Schmitt et al. (*6*), using a MAGPIX instrument (Luminex Corporation, Austin, TX, USA). Of the 180 cervical samples, 107 had been analyzed previously by using a Luminex 100 instrument (*5*), but there were no differences in sensitivity between the MAGPIX and Luminex 100 instruments. For comparison with the previous study (*5*), HPV types were classified as described by Muñoz et al. (*7*). Samples with values <30 for β-globin were excluded. Oral and cervical HPV prevalence was compared by using a 2-tailed Fisher exact probability test, and HPV16 concordance in oral and genital samples was measured by κ.

**Figure 1:**
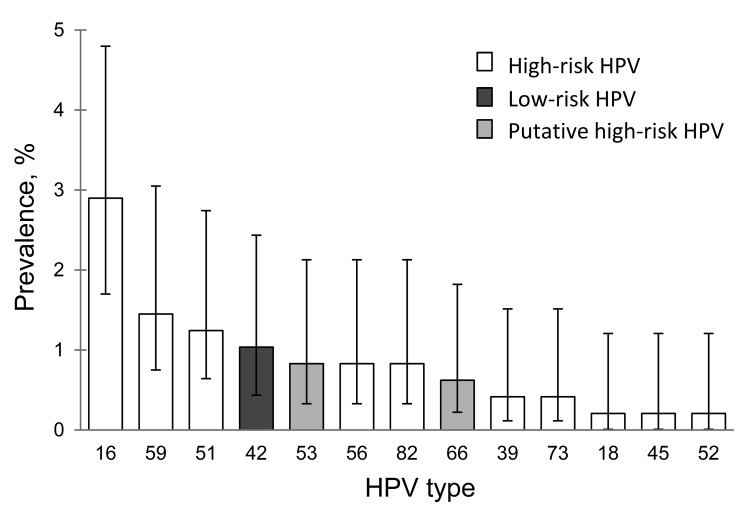
Prevalence of human papillomavirus (HPV) types in oral samples from 24 female youth with oral HPV infection, Stockholm, Sweden. The 4 most common HPV types were high-risk types HPV16 (2.9%, 95% CI 1.7%–4.8%), HPV59 (1.4%, 95% CI 0.7%–3.0%), and HPV51 (1.2%, 95% CI 0.6%–2.7%) and low-risk type HPV42 (1.0%, 95% CI 0.4%–2.4%).

Of the 490 oral samples, 7 were excluded because of insufficient material; of the remaining samples, 9.3% (45/483) were positive for HPV, 9.2% (37/401) in female and 9.8% (8/82) in male youth. Most HPV types detected in oral samples from both sexes were high-risk HPV (Table 1); >2 HPV types were found in 7 oral samples. Figure 1 shows the prevalence and 95% CIs for each HPV type. HPV16 was the most prevalent high-risk type detected (2.9%, 95% CI 1.7%–4.8%), and HPV42 was the only low-risk type detected (1.0%, 95% CI 0.4%–2.4%).

**Table 1:** HPV prevalence in oral and cervical samples from 24 female youth with oral HPV infection, Stockholm, Sweden*

HPV-positive categories	No. (%) samples	
	Oral	Cervical
All HPV types	45 (9.3)	129 (74.1)
All high-risk HPV types	35 (7.2)	113 (64.9)
HPV 16	14 (2.9)	66 (37.9)
HPV 18	1 (0.2)	25 (14.4)
Total no. samples	483	174

*HPV, human papillomavirus.

**Figure 2:**
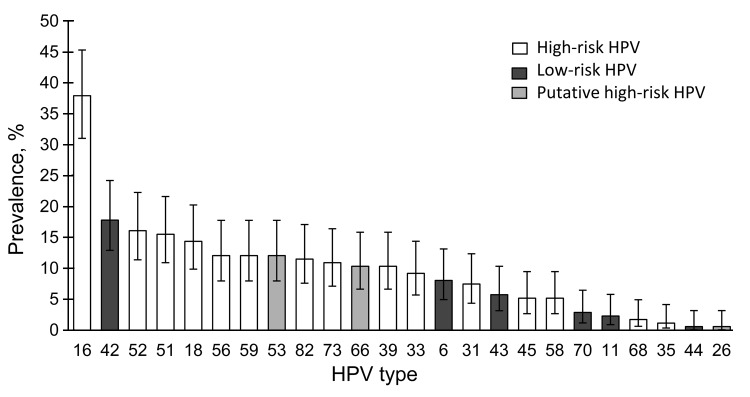
Prevalence of human papillomavirus (HPV) types in cervical samples from 24 female youth with oral HPV infection, Stockholm, Sweden. The 4 most common types were high-risk types HPV16 (37.9%, 95% CI 31.0%–45.3%); HPV52 (16.1%, 95% CI 11.4%–22.3%), and HPV51 (15.5%, 95% CI 10.9%–21.6%) and low-risk type HPV42 (17.8%, 95% CI 12.9%–24.2%).

The prevalence of oral and cervical HPV was compared for 174 female youth from whom oral and cervical samples were obtained together and contained sufficient material; cervical HPV was detected in 129 (74.1%). Most HPV types detected in the cervix were high-risk types. Figure 2 shows the prevalence and 95% CI for each HPV type. HPV16 was the most prevalent high-risk type detected (37.9%, 95% CI 31.0%–45.3%), followed by low-risk type HPV42 (17.8%, 95% CI 12.9%–24.2%).

Oral HPV infection was more frequent in female youth with (22/129, 17.1%) than without (2/45, 4.4%) cervical HPV infection (p = 0.043). HPV types commonly detected in the cervical tract were observed in the oral tract (Figure 1, Figure 2), but fewer HPV types were detected in the oral compared with the genital tract. In addition, median fluorescent intensity for HPV was lower in oral compared with genital samples (data not shown).

Among 24 female youth with oral HPV infection, 22 (91.7%) also had a cervical HPV infection (Table 2). Furthermore, for 20/22 (90.9%) of those who had concomitant oral and cervical HPV infection, oral HPV types were completely concordant with cervical HPV types, but the opposite was not true because there were more HPV types in general per cervical site (Table 2). For example, for female youth nos. 10 and 13, both of whom had several oral HPV types, all types in the oral tract were detected in the cervical tract but not vice versa (Table 2). Calculating κ 0.4400 for HPV16 in oral and cervical sites in the 24 persons with HPV-positive oral samples resulted in moderate agreement; slight agreement was obtained when calculating κ 0.1032 for the 129 female youth with HPV-positive cervical samples and 0.1345 for all 174 female youth.

**Table 2:** HPV types detected in oral and cervical samples from 24 female youth with oral HPV infection, Stockholm, Sweden*

Patient no.	Types detected from oral samples				Types detected from cervical samples		
	High risk	Putative high risk	Low risk		High risk	Putative high risk	Low risk
1	**16**				**16**, 18, 51, 82	53	
2	**16**				**16**, 33, 45	66	43
3	**16**				**16**, 18, 39		6, 42
4	**16**				**16**, 18, 31, 45, 73		42, 43
5	**16**				**16**, 31, 51, 52, 59	53	42
6	**16**				**16**, 51		
7	**16**				**16**		42
8	**16**				**16**		
9	**18**				**18**		6
10	**39, 51, 56, 59**				16, **39**, **51**, 52, **56**, **59**, 82	66	42
11	**51**				**51**		42, 43
12	**51**				**51**, 82		
13	**56, 59**				16, **56**, **59**		
14	**82**				18, 51, 52, 58, 68, **82**	53	42
15	**82**				16, 18, 51, 58, **82**	53	
16		**53**			33, 45, 51, 82	**53**	
17		**53**				**53**	42, 43
18			**42**				**42**
19			**42**		45, 56, 59	66	**42**
20			**42**		16, 33, 52, 56, 59	53	6, **42**
21	82				16, 33, 45, 73		11
22	73				16, 18, 52, 56	53, 66	
23	16						
24			42				

*Boldface indicates HPV types found in both oral and cervical samples from the same person. HPV, human papillomavirus.

## Conclusions

In this study, oral HPV prevalence was similar among male and female youth (9.3% vs. 9.8%) but higher for female youth with (17.1%) than without (4.4%) cervical HPV infection. Moreover, most female youth with oral HPV infection had cervical HPV infection with type concordance and dominance of HPV16.

Oral HPV prevalence in our cohort was comparable to that in other reports (*8*,*9*). Likewise, the higher oral HPV prevalence in female youth who had concurrent genital infection and the finding that most female youth with oral HPV also had genital HPV infection was similar to findings in the study by Giraldo et al. (*10*). Nevertheless, some differences in prevalence of HPV in cervical samples were seen between the study of Giraldo et al. (*10*) and our study; these differences could be attributed to the different populations, biologic sampling methods, and the assays used. We found a higher HPV type concordance between oral and cervical infections than that found in other studies (*11*). This difference may partly be because of differences between the populations included and the techniques used for detection of different HPV types (*11*).

We also found that most HPV types commonly found in cervical samples were detected in oral samples, which suggests no major differences between HPV types in the cervical and oral tracts. This finding is similar to some reports, but not all (*10*–*14*). The lower prevalence of oral than cervical HPV was consistent with previous findings (*10*–*14*); however, these findings may be underestimates because the continuous production of saliva causes viral DNA to be swallowed and disappear from the oral cavity. This may also partly, but not completely, explain the lack of or weaker HPV type concordance between cervical and oral locations.

Our study has several limitations. The sample size of our cohort is relatively small, particularly with regard to those with available concurrent cervical and oral samples. In addition, our cohort represents a sexually active group seeking assistance for sexually transmitted diseases or preventive measures, which means their overall HPV prevalence may be higher compared with that of other persons of the same age in Stockholm. We also do not have demographic and behavioral data for the participating youth, and these data could affect the calculation of risk for infection.

In conclusion, we found the prevalence of oral HPV infection, with dominance of HPV16, was similar for male and female youth, but among female youth, infection was more common for those who had co-occuring genital HPV infection, and most oral HPV types were also found in the genital tract. These data emphasize the importance of investigation to determine if the current HPV vaccines also prevent oral HPV infection.
